# Mitigating Psychological Problems Associated with the 2023 Wildfires in Alberta and Nova Scotia: Six-Week Outcomes from the Text4Hope Program

**DOI:** 10.3390/jcm13030865

**Published:** 2024-02-01

**Authors:** Gloria Obuobi-Donkor, Reham Shalaby, Belinda Agyapong, Raquel da Luz Dias, Vincent Israel Opoku Agyapong

**Affiliations:** 1 Department of Psychiatry, Faculty of Medicine, Dalhousie University, Halifax, NS B3H 4R2, Canada; gloria.ob@dal.ca (G.O.-D.); raquell.dias@nshealth.ca (R.d.L.D.); 2 Department of Psychiatry, Faculty of Medicine and Dentistry, University of Alberta, Edmonton, AB T6G 2B7, Canada; rshalaby@ualberta.ca (R.S.); bagyapon@ualberta.ca (B.A.)

**Keywords:** wildfire, psychological problems, Alberta, Nova Scotia, text messaging

## Abstract

**Background:** In 2023, wildfires led to widespread destruction of property and displacement of residents in Alberta and Nova Scotia, Canada. Previous research suggests that wildfires increase the psychological burden of impacted communities, necessitating population-level interventions. Cognitive Behavioural Therapy (CBT)-based text message interventions, Text4HopeAB and Text4HopeNS, were launched in Alberta and Nova Scotia, respectively, during the 2023 wildfire season to support the mental health of impacted individuals. **Objectives:** The study examines the effectiveness of Text4HopeNS and Text4HopeAB in alleviating psychological symptoms and improving wellbeing among subscribers. **Methods:** The study involved longitudinal and naturalistic controlled trial designs. The longitudinal study comprised subscribers who completed program surveys at baseline and six weeks post-enrolment, while the naturalistic controlled study compared psychological symptoms in subscribers who had received daily supportive text messages for six weeks (intervention group) and new subscribers who had enrolled in the program during the same period but had not yet received any text messages (control group). The severity of low resilience, poor mental wellbeing, likely Major Depressive Disorder (MDD), likely Generalized Anxiety Disorder (GAD), likely Post-Traumatic Stress Disorder (PTSD), and suicidal ideation were measured on the Brief Resilience Scale (BRS), the World Health Organization-5 Wellbeing Index (WHO-5), Patient Health Questionnaire 9 (PHQ-9), Generalized Anxiety Disorder 7 (GAD-7) scale, PTSD Checklist–Civilian Version (PCL-C), and the ninth question on the PHQ-9, respectively. The paired and independent sample *t*-tests were employed in data analysis. **Results:** The results from the longitudinal study indicated a significant reduction in the mean scores on the PHQ-9 (−12.3%), GAD-7 (−14.8%), and the PCL-C (−5.8%), and an increase in the mean score on the WHO-5, but not on the BRS, from baseline to six weeks. In the naturalistic controlled study, the intervention group had a significantly lower mean score on the PHQ-9 (−30.1%), GAD-7 (−29.4%), PCL-C (−17.5%), and the ninth question on the PHQ-9 (−60.0%) which measures the intensity of suicidal ideation, and an increase in the mean score on the WHO-5 (+24.7%), but not on the BRS, from baseline to six weeks compared to the control group. **Conclusions:** The results of this study suggests that the Text4Hope program is an effective intervention for mitigating psychological symptoms in subscribers during wildfires. This CBT-based text messaging program can be adapted to provide effective support for individuals’ mental health, especially in the context of traumatic events and adverse experiences such as those induced by climate change. Policymakers and mental health professionals should consider these findings when shaping strategies for future disaster response efforts, emphasizing the value of scalable and culturally sensitive mental health interventions.

## 1. Introduction

Wildfires are uncontrolled fires that spread rapidly through vegetation and natural landscapes, and have become a growing global concern in the context of climate change [1] due to their immediate physical damage to their environment and the long-term psychological implications on affected populations. Disasters affect over 43% of Canadians during their lifetime, with wildfires accounting for 20% of these disasters [2]. The 2023 wildfires in Canada were particularly severe, with almost 3 million hectares of vegetation burnt by 31 May, marking it as the most active spring season on record in western Canada [3]. On 6 May 2023, Alberta declared a state of emergency at the provincial level in response to a wildfire, leading to the evacuation of many residents from their homes [4]. Simultaneously, Nova Scotia experienced its most substantial wildfire on record, igniting on 27 May 2023 [5]. The wildfire resulted in extensive property destruction and the displacement of numerous residents [5].

The increasing intensity and duration of wildfire events underscore the importance of understanding the potential health effects and impacts on mental health and wellbeing [6]. The devastating impact of wildfires does not only cause environmental destruction but also has a profound effect on mental health, which has been well documented in various studies [7,8,9,10,11]. These adverse mental health effects include increased symptoms of anxiety, Post-Traumatic Stress Disorder (PTSD), and depression [7,8]. For instance, research on the 2016 Fort McMurray wildfire revealed significant PTSD and other mental health effects among students even 18 months after the event [8]. Furthermore, the high prevalence of anxiety symptoms six months after the wildfire in Fort McMurray underscores the significant psychological impact of such disasters [12]. Systematic reviews indicate that up to 40% of individuals who undergo a natural disaster, such as wildfires, may develop stress-related conditions [13].

In light of the psychological impact of wildfires, developing effective support and intervention strategies is essential to mitigate the long-term psychological impact on affected populations. Innovative, cost-efficient, technologically enabled, and easily scalable interventions, such as daily supportive SMS text messaging [14,15,16,17], may hold the key to the provision of support to impacted communities during and after wildfires. While the use of text messaging as an intervention in mental health has been explored in various contexts, its application in a wildfire-impacted population is a novel area for research [1,18]. Cognitive behavioural therapy (CBT) delivered via text messaging interventions has shown promise in reducing depressive symptoms [14,15,19], addressing mental health treatment gaps at the population level [20,21], and improving treatment outcomes for various psychological conditions. Furthermore, outreach psychological support services delivered through text messaging have effectively assisted individuals impacted by wildfires [22]. These findings suggest that supportive text message interventions have the prospect of being effective in reducing psychological symptoms in the aftermath of wildfires.

Moreover, the effectiveness of CBT-based text messaging interventions in addressing mental health conditions has been demonstrated in diverse populations [23,24], including individuals with comorbid diagnoses [25,26]. Additionally, studies have highlighted the importance of tailored and personalized messages delivered frequently and for longer duration in enhancing the efficacy of text messaging interventions [27] and significantly improving diverse health outcomes [28].

The increasing frequency and intensity of wildfires pose a significant threat to both the environment and human wellbeing. The 2023 wildfires in Canada, notably severe, prompt an urgent need to understand and address the immediate physical damage and long-term psychological implications on affected populations. The specific problem being addressed is the mental health impact of wildfires, with a focus on symptoms such as low resilience, quality of life, depression, anxiety, PTSD, and suicidal thoughts among individuals affected by the 2023 wildfires in Alberta and Nova Scotia.

Text4HopeNS and Text4HopeAB text messaging programs were implemented during the 2023 wildfires in Alberta and Nova Scotia, Canada. These programs are based on CBT principles to deliver mental health support to individuals during the recent wildfires. By understanding the impact of these interventions, policymakers can make informed decisions on the implementation and scaling of similar mental health support programs. The findings can inform the development of evidence-based strategies to address psychological distress, contributing to the enhancement of mental health services and policies for broader societal benefit. Hence, this study examines the severity of low resilience and quality of life, depression, anxiety, Post-Traumatic Stress Disorder (PTSD), and suicidal thoughts during the 2023 wildfires in Alberta and Nova Scotia, Canada. The study also seeks to assess the effectiveness of the Text4HopeNS and Text4HopeAB programs in alleviating symptoms of mental health conditions.

## 2. Methods

### 2.1. Study Design and Setting

This study adopted two designs: a longitudinal design and a naturalistic controlled trial design. The longitudinal study design assessed clinical outcomes among subscribers who completed both baseline and six-week surveys. The naturalistic controlled study compared two distinct study populations. The first group was an intervention group, which consisted of subscribers who received supportive text messages for six weeks and completed the sixth-week evaluation survey. The second group served as a control group, comprising subscribers who joined Text4HopeNS and Text4HopeAB in the same time frame and completed the enrolment (baseline) surveys but who were yet to receive any intervention in the form of supportive text messages.

The study was conducted in Alberta and Nova Scotia, Canada and the service was available to all residents. According to the 2021 census, Nova Scotia province had approximately 969,383 residents, while Alberta had 4,262,635 residents and occupied 661,848 square kilometres [29].

The choice of Alberta and Nova Scotia as research locations is due to the opportunity to collaborate with the regional health authorities in the launch of the Text4Hope program as an emergency psychological response initiative during the 2023 wildfire season.

### 2.2. Institutional Review Board Approval

This study adhered to the guidelines outlined in the Declaration of Helsinki and received approval from the Alberta Health Research Ethics Committee (Pro00086163) and the Research Ethics Board at Nova Scotia Health (REB file #1028254). Informed consent was implied after subscribers completed the online survey and submitted their responses.

### 2.3. Study Intervention

Text4HopeNS and Text4HopeAB are evidence-based tools that help individuals affected by wildfires and other traumatic experiences identify and adjust to negative thoughts, feelings, and behaviours during stressful times [30,31]. The Text4HopeNS and Text4HopeAB programs are self-subscribing programs, and individuals can sign up for the program by texting the word “HopeNS” for residents in Nova Scotia and “HopeAB” for residents in Alberta to a short code number to receive free unidirectional supportive SMS text messages for three months. Subscribers could opt out by texting “STOP” to the same short code number. The supportive text messages are delivered daily at 9:00 a.m. Mountain Time in Alberta and 9:00 a.m. Atlantic Time in Nova Scotia. These text messages were created by psychiatrists, psychologists, and mental health therapists based on CBT principles to promote self-care, resilience, social support, hope, and affirmation, as well as to help subscribers manage symptoms of stress, anxiety, and depression and improve mental wellbeing. Examples of the text messages included:

“No matter what setbacks you’ve faced or challenges that lie ahead, you can succeed if you have inner strength and stay focused. Have faith in yourself and success will be yours no matter what problems the wildfire throws at you.”

“There are two days in the week we should not worry about, yesterday and tomorrow. That leaves today, live for today. Thinking of the past or the future can be overwhelming for anyone facing a challenging situation or crisis.”

### 2.4. Data Collection

The study data were collected through an online survey completed by residents in Alberta and Nova Scotia, Canada using Research Electronic Data Capture (REDCap 13.7.1) software [32]. The study was conducted between 14 May and 23 September 2023. The initial Text4HopeNS and Text4HopeAB message welcomes subscribers and invites them to participate in a baseline survey. Subscribers receive follow-up surveys at six weeks, three months, and six months post-enrolment.

The survey questionnaire included a combination of sociodemographic information, gender, age, ethnicity, marital status, employment status, educational status, housing status, and clinical information, including various self-reported mental health symptoms.

### 2.5. Outcome Measures

The study’s primary outcome measures were the change in mean scores of the clinical scales from baseline to 6 weeks in the longitudinal study and between the intervention and control groups in the naturalistic controlled study. The clinical scales used included the Patient Health Questionnaire-9 (PHQ-9) [33], the Generalized Anxiety Disorder 7-item (GAD-7) [34], the PTSD Checklist–Civilian Version (PCL-C) [35], the Brief Resilience Scale (BRS) [36], the World Health Organization-5 Wellbeing Index (WHO-5) [37], and the ninth question on the PHQ-9 [33].

The PHQ-9 was used to measure the depressive symptoms of participants. The PHQ-9 is a nine-item measure on a four-point Likert scale from 0 (not at all) to 3 (nearly every day) with a total score of 0–4 normal, 5–9 mild, 10–14 moderate, 15–19 moderately severe, and 20–27 severe [33]. A score of 10 or higher indicates likely MDD. The reliability and validity of the tool have indicated it to have sound psychometric properties, and the internal consistency of the PHQ-9 has been shown to be high [33].

The GAD-7 scale was used to assess the likelihood of anxiety symptoms. The seven self-reported items are rated on a four-point Likert scale: 0 (not at all) to 3 (nearly every day), with a total score range of 0 to 21 [34]. A score of 10 or more was deemed to be likely GAD. The internal consistency and test–retest reliability of the GAD-7 was good and provided good criterion, construct, factorial, and procedural validity [38].

The PCL-C was used to assess likely PTSD symptoms. This is a self-report rating scale for PTSD comprising 17 items. The level of distress produced by each symptom is rated on a five-point Likert scale. A score ≥ 44 denotes likely PTSD, and a score below 44 indicates that PTSD is unlikely [35]. The PCL-C demonstrated good internal consistency and retest reliability and favourable patterns of convergent and discriminant validity [35,39].

The BRS assessed participants’ ability to recover from stress and was used to measure resilience in this study. A score ranging from 1.00 to 2.99 indicates low resilience, while a score ranging from 3.00 to 5.00 indicates normal to high resilience [36]. The BRS has good internal consistency, with Cronbach alphas ranging from 0.80 to 0.90 and test–retest reliability coefficients for a two-week interval of between 0.61 and 0.69 [36,40].

Wellbeing was assessed using the WHO-5 Wellbeing Index. The WHO-5 is a five-item measure on a six-point Likert scale [37,41]. The scale is scored by summing the values of the five responses, resulting in a raw score ranging from 0 to 25. A score of 0 indicates the lowest possible wellbeing, while 25 reflects the highest [37]. In this study, we employed a cut-off score below 13 as a criterion for poor mental wellbeing.

Participants were asked whether they had passive death wishes or thoughts of self-harm in the last two weeks. This was assessed via the ninth question of the PHQ-9 scale [33].

### 2.6. Hypotheses

For the longitudinal study, it is hypothesized that subscribers who received the intervention and completed surveys at the two time points would have at least 20% lower mean scores on PHQ-9, GAD-7, and PCL-C and higher mean scores on the BRS and WHO-5 at six weeks compared with their baseline scores. For the naturalistic controlled study, it is hypothesized that subscribers who have received the daily supportive text messages for six weeks (intervention group) would have at least 20% lower mean scores on the PHQ-9, GAD-7, and PCL-C scales and higher mean scores on the BRS and WHO-5 scales compared to subscribers who were yet to receive the intervention (control group). These hypotheses are based on outcomes of previous randomized controlled trials, and population-level studies which reported a between 20% and 50% reduction in psychological symptoms among participants who received daily supportive text message interventions for six weeks or three months [14,15,24,42,43,44,45].

### 2.7. Sample Size Considerations

For the longitudinal study, to achieve a power of 80% and a level of significance of 5% (two-sided) for detecting an effect size of 0.3 between pairs, a sample size of 90 would be needed [46]. For the naturalistic controlled study, assuming a pooled standard deviation of 5 units, the study would require a sample size of 99 for each group (i.e., a total sample size of 198, assuming equal group sizes), to achieve a power of 80% and a level of significance of 5% (two-sided), for detecting a true difference in means between the intervention and the control group of −2 (i.e., 8–10) units [47].

### 2.8. Statistical Analysis

SPSS for Windows, version 28 (IBM Corporation, Armonk, NY, USA) [48] was used for the data analysis.

For the longitudinal study, the paired sample *t*-test was used to compare the mean scores of the clinical scales (BRS, WHO-5, PHQ-9, GAD-7, PCL-C, and the ninth question of the PHQ-9 which measures the intensity of suicidal ideation) from baseline to six weeks. We assessed the potential generalizability of the finding in our longitudinal study for all subscribers of Text4Hope who completed the baseline survey by using the Chi square test to assess if there are differences in the sociodemographic and clinical characteristics between subscribers who completed both baseline and six-week follow-up questionnaires and subscribers who completed the baseline survey.

For the naturalistic controlled study, the Chi square test and the independent sample *t*-test were, respectively, used to compare the sociodemographic characteristics and the mean scores of the clinical scales (BRS, WHO-5, PHQ-9, GAD-7, PCL-C, and the ninth question of the PHQ-9 which measures the intensity of suicidal ideation) between subscribers who had received daily supportive text messages for six weeks (intervention group) to the new subscribers who had enrolled in the program during the same designated two-month time frame but had not yet received any text messages (control group). No imputations were conducted for missing data, and the analysis and reporting were based on the actual number of responses received for each variable.

## 3. Results

### 3.1. Longitudinal Study Results

**Figure 1:**
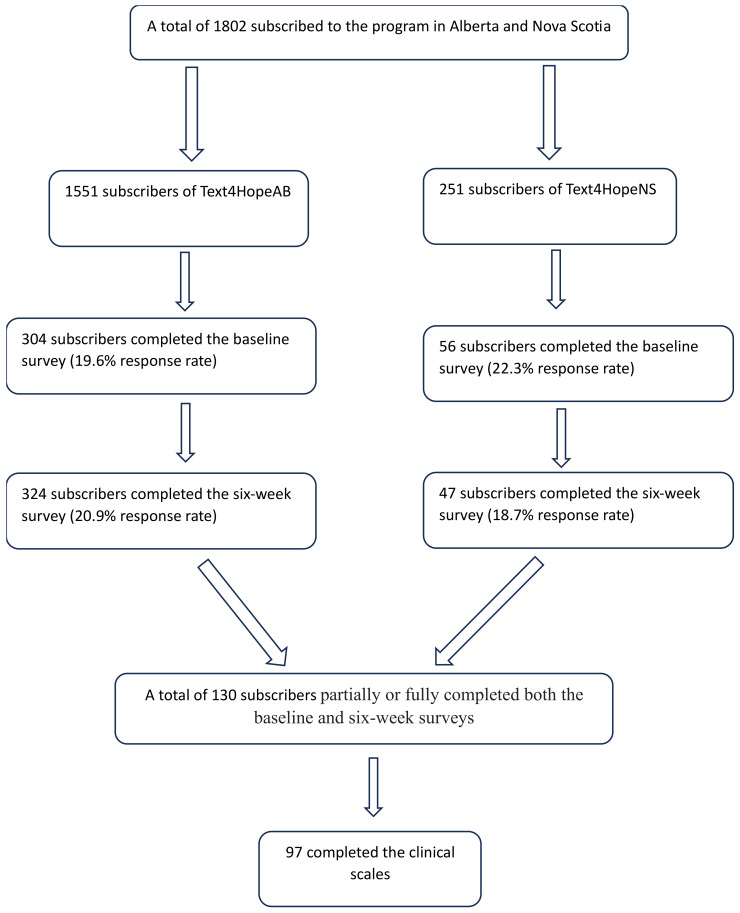
Study flow chart.

Figure 1 shows the study flow in Alberta and Nova Scotia. Of the 360 subscribers who completed the baseline survey, 304 were from Alberta and 56 from Nova Scotia. The response rates for the baseline were 19.6% and 22.3% in Alberta and Nova Scotia, respectively with an overall baseline response rate of 20%, while 130 subscribers partially or fully completed both the baseline and six-week surveys with 97 completing the clinical scales.

Appendix A illustrates the sociodemographic characteristics of all the study participants. The majority of the respondents were between 31 and 65 years old (371, 87.5%), female (356, 84.0%), Caucasian (356, 84.0%), had post-secondary education (350, 82.5%), were married, cohabiting or partnered (228, 53.9%), employed (267, 63.0%), and owned a house (274, 64.6%).

Table 1 illustrates the distribution of demographic characteristics of study participants who completed both the baseline and six-week surveys and participants who completed only the baseline survey. The results suggest that subscribers who completed both the baseline and six-week surveys had no marked differences between their demographic and clinical characteristics except their age, where a greater percentage of subscribers were between the ages of 31 and 50 years in the former compared to the latter.

Table 2 represents the changes in mean scores for the primary outcome variables for the Text4HopeNS and Text4HopeAB programs of participants who completed both the baseline and six-week scales.

Results from Table 2 indicate that there was a statistically significant reduction in the mean scores on the PHQ-9, GAD-7, and PCL-C and an increase on the WHO-5 Wellbeing Index from baseline to six weeks. There was an increase in the mean score on the BRS but it was not statistically significant.

### 3.2. Naturalistic Controlled Study Results

The naturalistic controlled trial results reported data from two distinct groups of participants: the intervention group (who received supportive messages) and the control group (who were yet to receive supportive text messages). The total number of subscribers who completed the surveys at baseline or at six weeks was 226; 52 (23%) in the control group and 174 (77%) in the intervention group.

Table 3 illustrates the demographic characteristics of subscribers in the intervention group and the control group at the time of their enrolment in the program.

Results from Table 3 suggest that the demographic characteristics of subscribers in the two groups were similar.

Table 4 represents the difference in mean scores for the primary outcome variables for Text4HopeNS and Text4HopeAB programs subscribers who completed the six-week scales (intervention group) and the baseline scores for new subscribers (control group) during the same time frame.

As illustrated in Table 4, there was a statistically significant difference between the intervention and control groups for all the clinical measures (*p* < 0.001) with medium effect sizes. There was a trend to finding a significant difference in the mean score on the BRS for the intervention group (mean 17.3, SD 4.9) and the control group (mean 16.0, SD 5.1; t_375_ = −1.75; *p* = 0.08). Finally, the mean scores of suicidal ideation or thoughts of self-harm in the intervention group (mean 0.27, SD 0.6) were significantly lower than in the control group (mean 0.65, SD 1.0; t_359_ = 3.85; *p* < 0.001), with an effect size of 0.57 (95% CI, 0.19–0.59). Suicidal ideation or thoughts of self-harm recorded the highest percentage difference of 58.5%.

## 4. Discussion

To the best of our knowledge, the Text4HopeNS and Text4HopeAB programs are the first CBT-based text messaging programs implemented at the population level and evaluated in any country in the world, to provide mental health support to individuals during wildfires. Results from the longitudinal study show that the mean scores on the WHO-5 Wellbeing Index, PHQ-9, GAD-7, and PCL-C and the ninth question of the PHQ-9 which measures the intensity of suicidal ideation, were statistically significantly improved at six weeks compared to their corresponding baseline scores, except on the BRS. Overall, these results suggest there were clinical improvements in subscribers’ clinical conditions.

In the naturalistic study, the mean scores on the WHO-5, PHQ-9, GAD-7, PCL-C, and the ninth question of the PHQ-9 were significantly lower in the intervention group compared to the control group. These results suggest that Text4HopeNS and Text4HopeAB were effective in reducing the severity of psychological conditions and improving wellbeing, but not resilience, during wildfires.

Our study results agree with various studies highlighting diverse approaches to addressing mental health conditions, improving relapse outcomes, and preventing acute episodes [14,49,50]. The literature has yet to extensively explore the use of text message interventions in the context of wildfires [51]. However, evidence suggests that text messaging interventions have effectively reduced psychological distress and improved mental wellbeing in various populations facing mental health challenges [20,24,52]. Agyapong et al. (2020) demonstrated the potential of text messaging interventions in reducing the psychological treatment gap for mental health patients, highlighting the affordability, availability, and scalability of text messaging as a supportive tool [17]. Similarly, a study by the same research group reported that subscribers felt more hopeful, in charge of managing their mental health issues, and connected to a support system through daily supportive text messages, ultimately improving their overall mental wellbeing [14,17,53].

Various theories support our study, for example, the Text4Mood [52,54], Text4PTSI [15], and the Text2Quit [55] Supportive Text Messaging Programs, and highlights the impact of supportive text messaging programs on psychological problems. These studies emphasize the potential of text messaging interventions in addressing psychological issues, providing a theoretical basis for the effectiveness of such programs in mitigating the psychological impact of the wildfire season.

Findings from this study show a reduction in the intensity of suicidal ideation in both the longitudinal and naturalistic studies, with the controlled study suggesting a 60% significant reduction in intensity of suicidal ideation in the intervention group. This result is a significant finding in the context of suicide prevention in the context of climate change and associated mental health burden. The results of this study are consistent with previous research on technology-enhanced suicide prevention interventions, which have shown significant reductions in the prevalence and intensity of suicidal ideation [56,57]. This reduction in intensity aligns with the assertion that suicidal ideation must become an essential intervention target [58]. Furthermore, the use of text messaging as a supportive tool has been associated with fewer suicide attempts and reduced suicidal ideation in previous clinical trials [57,59,60,61]. The reduction in the intensity of suicidal ideation is particularly noteworthy given the associations of slower psychomotor speed and set-shifting with suicidal ideation [62]. This suggests that the intervention may have positively impacted cognitive and psychomotor functions related to suicidal ideation. Moreover, improvement in negative emotions mediates the reduction in suicidal ideation intensity, which aligns with the potential emotional impact of the supportive text message intervention [63]. The reduction in suicidal ideation intensity is in line to promote recovery from suicidal ideation through the development of protective factors [64]. Other literature has shown that text interventions may effectively reduce suicidal ideation with positive thinking, especially for individuals who do not seek support from other sources [65]. Although crisis helplines can provide mental health support to individuals during traumatic events such as wildfires, Text4HopeNS and Text4HopeAB can provide convenient, confidential, and continuous support simultaneously to thousand individuals at risk for suicide, and improve their mental wellbeing by mitigating common mental health symptoms [66] as evidenced by the study results.

The heightened occurrence of mental health disorders among subscribers who have not received text message intervention agrees with various studies which suggest that individuals who do not receive support during wildfires are at a higher risk of experiencing negative mental health consequences compared to individuals who receive support. For instance, researchers in the literature have found that individuals with high resilience scores after experiencing a wildfire had lower scores on screening measures for mental health disorders, including PTSD, depression, and anxiety, and high resilience increased self-esteem and mental wellbeing [1,6,67]. Furthermore, exposure to smoke from wildfires may have mental health impacts, particularly in episodes of chronic and persistent smoke events, with evidence pointing to an increased prevalence of mental health and addiction conditions in patients attending clinics after wildfires [68].

The severity of mental health conditions in this study was high in the control group for the naturalistic study compared to the intervention group. This finding is consistent with other studies which reported associations between wildfires and adverse mental health effects in individuals, including increased symptoms of PTSD, depression, and anxiety [6,7]. The absence of support during wildfires exacerbates the risk of developing mental health issues, emphasizing the importance of providing timely and effective health support to mitigate these adverse outcomes. The reduction in severity of low resilience, poor mental wellbeing, depression, anxiety, and PTSD in this study agrees with a study conducted during the COVID-19 pandemic [68]. The study found that the text message intervention resulted in about a 25% reduction in mean scores on standardized rating scales for anxiety and depression in the third month compared to baseline [69].

Additionally, a cross-sectional study reported that 77% of subscribers to a similar text message program indicated that the daily supportive text messages helped them manage their depression and anxiety [69]. Positive results are recorded when text message interventions are targeted to minimize a particular mental health condition [19]. This provides a potential explanation as to why the Text4HopeNS and Text4HopeAB programs improved subscribers’ mental wellbeing. The need to optimize the quality of mental health interventions to provide better mental health support for populations impacted by wildfires has been highlighted [70]. One study demonstrated that participants in a computer-based preventive intervention experienced improved mental health outcomes relative to those who received a placebo, emphasizing the positive impact of supportive interventions on mental health [71]. However, the scalability of some computer-based interventions could be challenging in situations of natural disasters such as wildfires. A comparative control study between a wildfire-affected community and a control community which has not experienced wildfires reported a statistically significant increase in depression and thoughts of suicidal symptoms among the community impacted by the wildfire [72], suggesting the need for evidence-based, cost-effective, and easily scalable interventions to support wildfire-impacted communities. Although the supportive text messages were delivered as an add-on to individuals’ conventional therapeutic approaches, most interventions may exhibit their primary effects after or within six weeks of the interventions [44,73,74]. Therefore, it is commonly recommended to assess changes in symptoms after this timeframe [44,75]. Within six weeks of providing supportive messages to subscribers, the program demonstrated its ability to improve the individual’s mental wellbeing.

Furthermore, a study in California revealed the feasibility and efficacy of a public mobile app to minimize symptoms of post-disaster distress in wildfire survivors, indicating the potential for mobile interventions in this context [76]. Additionally, a study conducted during the Fort McMurray wildfire suggests that supportive text messaging, along with rapid identification and referral of those most affected, could help to mitigate the mental health effects of wildfires [68].

The results also suggest that the Text4HopeNS and Text4HopeAB programs had the least impact on the resilience level of subscribers in the longitudinal study, which suggests that the effectiveness of a text messaging intervention may vary across different settings and health conditions [77]. Thus, it also emphasizes the need to consider the specific context when evaluating the effectiveness of text message interventions and highlights the need for further research to understand the factors influencing the outcome. Text4HopeNS and Text4HopeAB have undeniably provided immediate relief to residents during a challenging period. Their accessibility and scalability make the programs a valuable tool in the broader mental health landscape.

## 5. Limitations

The study has limitations that should be acknowledged. The naturalistic controlled study design has inherent limitations owing to the real-world implementation of the program. Establishing a separate group without the intervention proved impractical due to the program’s nature, where randomization is deemed unethical, given the potential harm to individuals needing support during the wildfire. Secondly, clinical scales used to report symptoms were self-reported, and although the scales are validated and standardized, they are nonetheless non-diagnostic. Finally, the study’s strength may be minimized due to the relatively small effect sizes; however, interventions without a therapist usually record smaller effect sizes than therapist-involved studies [17,78,79]. In light of these limitations, the study contributes invaluable perspectives and substantiating evidence regarding the efficacy of the Text4Hope programs in alleviating the mental health burden during wildfires.

## 6. Conclusions and Implications for Policy and Practice

The results of this study suggest that the Text4Hope program is an effective intervention for mitigating psychological symptoms in subscribers during wildfires. Evidence from this study underscores the significant impact of supportive text message interventions in mitigating mental health conditions during wildfires. The evidence suggests that timely and targeted text-based support can contribute to substantial reductions in mental health symptoms, providing a valuable resource for individuals facing psychological challenges associated with such environmental disasters. The positive outcomes observed highlight the potential of innovative and accessible interventions, such as Text4HopeNS and Text4HopeAB, to play an important role in promoting mental wellbeing during times of crisis. The findings also resonate with the broader goal of promoting recovery from suicidal ideation and addressing negative emotions associated with suicidal ideation. There is, however, the need for further research to refine and optimize the effectiveness of such interventions for broader application in disaster-stricken communities. These findings add to the expanding wealth of knowledge regarding the efficacy of supportive text message interventions and present a promising avenue for provincial and national governments to address the mental health needs of individuals and populations in the face of climate change-induced environmental adversities.

## Figures and Tables

**Table 1:** Distribution of demographic characteristics of study participants who completed both the baseline and six-week surveys and participants who completed only the baseline survey.

Variable	Survey Completed		Chi^2^/Fisher’s Exact ^a^/*t*-Test (df)	*p*-Value
	Both Baseline and Six-Week Surveys *n* (%) *n* = 97 *	Baseline Survey Only *n* (%) *n* = 263		
Province				
Alberta	80 (82.5)	224 (85.2)	0.4	0.6
Nova Scotia	17 (17.5)	39 (14.8)		
Age (years)				
≤30	7 (7.2)	39 (14.9)	23.7	<0.001
31–50	24 (24.7)	120 (45.8)		
51–65	50 (51.5)	81 (30.9)		
>65	16 (16.5)	22 (8.4)		
Gender				
Male	14 (14.4)	35 (13.4)	0.6	0.8
Female	82 (84.5)	221 (84.4)		
Other	1 (1.0)	6 (2.3)		
Ethnicity				
Caucasian	86 (88.7)	215 (82.1)	2.3 ^a^	0.7
Indigenous	4 (4.1)	16 (6.1)		
Asian	4 (4.1)	12 (4.6)		
Black/Hispanic	1 (1)	8 (3.1)		
Other	2 (2.1)	11 (4.2)		
Education				
Elementary school	1 (1.0)	6 (2.3)	0.8	0.7
High school	14 (14.4)	43 (16.4)		
Post-secondary (college, trade school, university, or postgraduate study)	82 (84.5)	213 (81.3)		
Relationship status				
Married/Partnered/Common-Law/Cohabiting	47 (48.5)	148 (56.7)	6.4 ^a^	0.2
Single	24 (24.7)	72 (27.6)		
Separated or divorced	17 (17.5)	29 (11.1)		
Widowed	6 (6.2)	7 (2.7)		
Other	3 (3.1)	5 (1.9)		
Employment status				
Student	3 (3.1)	12 (4.6)	3.9	0.3
Employed	57 (58.8)	168 (64.1)		
Unemployed	15 (15.5)	45 (17.2)		
Retired	22 (22.7)	37 (14.1)		
Housing status				
Own home		167 (63.7)	1.2	0.6
Renting accommodation		65 (24.8)		
Live with family and friends		30 (11.5)		
BRS	Mean Score (SD)			
	*n* = 97	*n* = 216		
	2.7 (0.9)	2.8 (0.8)	−0.4 (311)	0.7
WHO-5 Wellbeing Index	Mean Score (SD)			
	*n* = 97	*n* = 213		
	9.6 (5.1)	10.3 (5.2)	−1.1 (308)	0.3
PHQ-9	Mean Score (SD)			
	*n* = 96	*n* = 203		
	11.3 (6.4)	11.4 (6.6)	0.2 (304)	0.8
GAD-7 scale	Mean Score (SD)			
	*n* = 96	*n* = 207		
	9.5 (5.5)	9.6 (5.8)	−0.04 (301)	0.9
PTSD PCL-C	Mean Score (SD)			
	*n* = 96	*n* = 203		
	43.2 (16.2)	41.3 (16.3)	0.7 (297)	0.4

* Total is variable according to the completed responses provided. ^a^ Fisher’s Exact

**Table 2:** Changes in mean scores of BRS, WHO-5, PHQ-9, GAD-7, and PCL-C from baseline to six weeks for Text4HopeNS and Text4HopeAB subscribers.

Measure	*n* *	Mean Scores		Mean Difference (95% CI)	% Change from Baseline	*p*-Value	*t*-Test	Effect Size (Hedges’ *g*)
		Baseline Mean (SD)	6-Week Mean (SD)					
BRS total score	97	2.7 (0.9)	2.8 (0.8)	−0.85 (−0.20–0.03)	+3.7	0.158	−1.42	0.12
WHO-5 total score	94	9.5 (5.1)	11.6 (5.2)	−2.09 (−3.01 to −1.18)	+22.1	0.000	−454	0.41
PHQ-9 total score	93	11.7 (6.5)	10.3 (6.2)	1.44 (0.48–2.4)	−12	0.004	2.97	0.22
GAD-7 total score	92	9.6 (5.4)	8.2 (5.3)	1.42 (0.53–2.32)	−14.6	0.002	3.15	0.26
PCL-C total score	90	43.6 (16.4)	41.1 (16.3)	2.52 (0.53–4.5)	−5.7	0.014	2.51	0.15
Suicidal ideation or thoughts of self-harm	93	0.29 (0.6)	0.26 (0.5)	−0.03 (−0.75–0.14)	−10.3	0.55	0.59	0.05

* Total is variable according to the completed responses provided.

**Table 3:** Distribution of the demographic characteristics of subscribers in the intervention and control groups.

Variable	Control Group *n* = 52	Intervention Group *n* = 174	Total *n* = 226	Chi^2^/Fisher’s Exact ^b^	*p*-Value
Age (years)					
≤30	10 (19.2)	16 (9.2)	26 (11.5)	7.1	0.07
31–50	20 (38.5)	52 (29.9)	72 (31.9)		
51–65	17 (32.7)	76 (43.7)	93 (41.2)		
>65	5 (9.6)	30 (17.2)	35 (15.5)		
Gender					
Male	10 (19.2)	28 (16.1)	38 (16.8)	0.6	0.8
Female	41 (78.8)	143 (82.2)	184 (81.4)		
Other	1 (1.9)	3 (1.7)	4 (1.8)		
Ethnicity					
Caucasian	43 (82.7)	150 (86.2)	193 (85.4)	1.9 ^b^	0.72
Indigenous	2 (3.8)	6 (3.4)	8 (3.5)		
Asian	4 (7.7)	9 (4.0)	13 (5.8)		
Black/Hispanic	3 (5.8)	6 (3.4)	9 (4.0)		
Other	0 (0.0)	3 (1.7)	3 (1.3)		
Education					
Elementary school	1 (1.9)	1 (0.6)	2 (0.9)	2.2 ^b^	0.28
High school	11 (21.2)	28 (16.1)	39 (17.3)		
Post-secondary (college, trade school, university, or postgraduate study)	40 (76.9)	145 (83.3)	185 (81.9)		
Relationship status					
Married/Partnered/Common-Law/Cohabiting	28 (53.8)	87 (50.0)	115 (50.9)	3.6 ^b^	0.49
Single	15 (28.8)	42 (24.1)	57 (25.2)		
Separated or divorced	6 (11.5)	32 (18.4)	38 (16.8)		
Widowed	1 (1.9)	10 (5.7)	11 (4.9)		
Other	2 (3.8)	3 (1.7)	5 (2.2)		
Employment status					
Student	3 (5.8)	5 (2.9)	8 (3.5)	4.3 ^b^	0.22
Employed	29 (55.8)	107 (61.5)	136 (60.2)		
Unemployed	11 (21.2)	21 (12.1)	32 (14.2)		
Retired	9 (17.3)	41 (23.6)	50 (22.1)		
Housing status					
Own home	29 (55.8)	116 (66.7)	145 (64.2)	2.2	0.34
Renting accommodation	16 (30.8)	38 (21.8)	54 (23.9)		
Live with family and friends	7 (13.5)	20 (11.5)	27 (11.9)		

^b^ Fisher’s Exact.

**Table 4:** Independent sample *t*-test comparing the mean scores on clinical scales for the intervention and control groups.

Measure	*n*	Mean Scores		*n*	Mean Difference between Groups (95% CI)	% Difference between Groups	*p*-Value	*t*-Test	Effect Size (Hedges’ *g*)
		Intervention Group (SD)	Control Group (SD)						
BRS total score	327	17.3 (4.9)	16.0 (5.1)	50	+1.3		0.08	−1.75	0.26
					(−2.7–0.16)	+8.1			
WHO-5 total score	315	11.9 (4.9)	9.6 (5.4)	49	+2.38		0.002	−3.07	0.46
					(−3.9–−0.86)	+24.7			
PHQ-9 total score	321	9.2 (6.1)	13.1	49	−3.94		<0.001	4.2	0.64
			(6.3)		(2.09–5.78)	−30.1			
GAD-7 total score	307	7.8 (5.5)	11.1 (5.6)	48	−3.26		<0.001	3.76	0.6
					(1.55–4.96)	−29.4			
PCL-C total score	295	38.1 (16.1)	46.2 (14.7)	47	−8.09		0.001	3.22	0.5
					(3.15–13.0)	−17.5			
Suicidal ideation or thoughts of self-harm	312	0.27 (0.6)	0.65 (1.0)	49	−0.39		<0.001	3.85	0.57
					(0.19–0.59)	−60.0			

## Data Availability

Upon a reasonable request, the study data supporting the conclusions of this article can be obtained from the corresponding author.

## Appendix A. Distribution of Demographic Characteristics of Study Participants

**Variable**	**Province**		**Total** ***n* = 424**
	**Alberta *n* (%)** ***n* = 358**	**Nova Scotia *n* (%)** ***n* = 66**	
Age (years)			
≤30	46 (12.8)	7 (10.6)	53 (12.5)
31–50	137 (38.3)	31 (47)	168 (39.6)
51–65	133 (37.2)	22 (33.3)	155 (36.6)
>65	42 (11.7)	6 (9.1)	48 (11.3)
Gender			
Male	47 (13.1)	12 (18.2)	59 (13.9)
Female	304 (84.9)	52 (78.8)	356 (84.0)
Other	7 (2.0)	2 (3.0)	9 (2.1)
Ethnicity			
Caucasian	299 (83.5)	57 (86.4)	356 (84.0)
Indigenous	21 (5.9)	0 (0.0)	21 (5.0)
Asian	20 (5.6)	0 (0.0)	20 (4.7)
Black/Hispanic	8 (2.2)	5 (7.6)	13 (3.1)
Other	10 (2.8)	4 (6.1)	14 (3.3)
Education			
Elementary school	5 (1.4)	2 (3.0)	7 (1.7)
High school	60 (16.8)	7 (10.6)	67 (15.8)
Post-secondary (college, trade school, university, or postgraduate study)	293 (81.8)	57 (86.4)	350 (82.5)
Relationship status			
Married/Partnered/Common-Law/Cohabiting	186 (52.1)	42 (63.6)	228 (53.9)
Single	94 (26.3)	17 (25.8)	111 (26.2)
Separated or divorced	56 (15.7)	3 (4.5)	59 (13.9)
Widowed	14 (3.9)	2 (3.0)	16 (3.8)
Other	7 (2.0)	2 (3.0)	9 (2.1)
Employment status			
Student	12 (3.4)	5 (7.6)	17 (4.0)
Employed	224 (62.6)	43 (65.2)	267 (63.0)
Unemployed	59 (16.5)	9 (13.6)	68 (16.0)
Retired	63 (17.6)	9 (13.6)	72 (17.0)
Housing status			
Own home	230 (64.2)	44 (66.7)	274 (64.6)
Renting accommodation	85 (23.7)	17 (25.8)	102 (24.1)
Live with family and friends	43 (12.0)	5 (7.6)	48 (11.3)
Other—categories are not included in the survey options.
